# Host-Microbiota Interactions in Ileum and Caecum of Pigs Divergent in Feed Efficiency Contribute to Nutrient Utilization

**DOI:** 10.3390/microorganisms8040563

**Published:** 2020-04-14

**Authors:** Henry Reyer, Michael Oster, Ursula M. McCormack, Eduard Muráni, Gillian E. Gardiner, Siriluck Ponsuksili, Peadar G. Lawlor, Klaus Wimmers

**Affiliations:** 1 Institute for Genome Biology, Leibniz Institute for Farm Animal Biology (FBN), Wilhelm-Stahl-Allee 2, 18196 Dummerstorf, Germany; reyer@fbn-dummerstorf.de (H.R.); oster@fbn-dummerstorf.de (M.O.); murani@fbn-dummerstorf.de (E.M.); ponsuksili@fbn-dummerstorf.de (S.P.); 2 Animal and Grassland Research and Innovation Centre, Teagasc Pig Development Department, Moorepark, Fermoy, Co. Cork P61 C996, Ireland; Ursula.Mccormack@dsm.com (U.M.M.); peadar.lawlor@teagasc.ie (P.G.L.); 3 Department of Science, Waterford Institute of Technology, Waterford, Co. Waterford X91 K0EK, Ireland; GGARDINER@wit.ie; 4 Faculty of Agricultural and Environmental Sciences, University Rostock, 18059 Rostock, Germany

**Keywords:** residual feed intake, gene expression, caecum, ileum

## Abstract

The composition of the intestinal microbiota plays an important role in the digestion and utilization of nutrients and for gut health. Low-fiber diets stimulate digestion and absorption processes, predominantly in the upper region of the gastrointestinal tract, thereby increasing the conversion of feed into body weight. As a consequence, the chemical composition of digesta after duodenal and jejunal absorption processes and passage has a limited complexity affecting colonization and molecular profiles of enterocytes in the hind gut. To decipher ileal and caecal microbial ecosystems and host transcriptional profiles that are beneficial for effective use of the remaining nutrients, pigs differing in feeding efficiency were studied. Biological functions that were consistently enriched at both the gene and microbiota levels comprise immunity-related processes, which ensure the integrity of the gastrointestinal tract. In addition, the differential abundance of certain genera, including *Rothia*, *Subdoligranulu*, *Leeia* and *Cellulosilyticum*, reflects the establishment of a microbial profile that supports the digestion of endogenously indigestible dietary components in highly feed-efficient pigs. Overall, the results indicate the potential to promote these beneficial functions and further improve feed efficiency through manipulation of dietary and probiotic strategies.

## 1. Introduction

Over the last decade, intestinal and faecal microbiota in pigs have been analyzed with the emphasis on their effect on animal health and pig growth, including in particular traits related to feed efficiency (e.g., [1,2]). The composition of the microbiota plays an important role in the degradation of fiber, the education/programming of the immune system, the suppression of pathogens by competition and secretion of antimicrobial compounds, and the supply of energy equivalents and vitamins such as butyrate and vitamin K_2_. As well as developing strategies to modulate the microbiota composition and fermentation processes by feeding, e.g., prebiotics and probiotics, a major research focus is to elucidate the host-microbiota interaction and the establishment and differentiation of specific enterotypes. Microbial colonization is influenced by diet, age, and the segment of the gastrointestinal tract sampled, as well as their interaction [3]. Consequently, the spatiotemporal abundance of several microbial taxa has been correlated with feed efficiency traits in pigs [2,4]. In particular, microbes, which enable the host to use dietary proteins and carbohydrates efficiently, and those that promote intestinal health, are proposed as the most important contribution to increasing feed efficiency [4,5].

Genetic factors of the host are thought to strongly influence the abundance of distinct bacterial species or clusters of related taxa [6]. Gene expression profiling has been shown to be a very effective tool for investigating the molecular dialogue between intestinal epithelial cells and microorganisms [7,8]). These studies revealed a number of host genes expressed in the gastrointestinal mucosa, which contribute to the repertoire of the host to shape intestinal microbiota composition. Moreover, the transcriptome of intestinal cells determines intestinal morphology (e.g., villi and crypt size), intestinal barrier function (e.g., expression of mucin and tight junction encoding genes), nutrient digestibility, and absorption processes (expression of intestinal nutrient transporters) [9,10,11,12]. Other investigations provide evidence that these regulations are bidirectional and that microbial taxa can alter the host transcriptome. About ten percent of the host’s transcriptome is expected to be microbially regulated, mainly affecting genes involved in immunity, cell proliferation, and metabolism [13]. At the local level, this might help create a favorable intestinal environment for certain microbes, whereas at the systemic level effects on the brain are also assumed [14].

The current study examined the abundance of microbial taxa in ileum and caecum digesta and the transcriptomic profiles of the corresponding intestinal mucosa of pigs classified as exhibiting low and high feed efficiency (expressed as residual feed intake, RFI). The contribution of the ileum and caecum to feed efficiency is of particular interest considering the use of low-fiber concentrate diets in pig production, which stimulate digestion and absorption processes predominantly in the upper part of the gastrointestinal tract. Through the integration of information on affected host pathways and predicted functions of commensal microbiota, the objective here was to identify gene clusters that correlate with the occurrence of specific microbial taxa in pigs divergent for feed efficiency. Consequently, it may then be possible to use host-microbiota interactions in exploiting intestinal microbial composition as a predictive parameter for feed efficiency.

## 2. Materials and Methods

### 2.1. Animals and Tissue Sampling

The animal trial was approved by the animal ethics committees of Teagasc (TAEC9/2013) and Waterford Institute of Technology (13/CLS/02) and was licensed by the Irish Health Products Regulatory Authority (AE1932/P004). The study procedures complied with the European Union Directive 2010/63/EU on the protection of animals used for scientific purposes. The trial group comprised 138 pigs from a Large White x Landrace cross, and was described in detail by McCormack et al. [15]. In brief, after weaning at day 28, intact litters were grouped in pens of 10–13 pigs/pen. Pens were equipped with feed intake recording equipment (FIRE) feeders (Schauer Agrotronic, Wels, Austria) and pigs had ad libitum access to feed and water. Based on phenotypic data collected weekly, namely body weight, back fat and muscle depth (ultrasonic measurement), residual feed intake (RFI) was calculated at 120 day of age. The most divergent animals within litter and sex were selected and classified as low RFI (high feed efficient) and high RFI (low feed efficient) pigs. Slaughter and tissue collection of 20 high and 20 low RFI pigs took place around day 134 of life. Intestinal samples were taken 15 cm proximal to the ileo-cecal junction (ileum) and from the terminal tip of caecum. The digesta was taken from the 5 cm long segment in a sterile tube. The mucosa was scraped off with a glass slide after washing with phosphate-buffered saline. Samples were snap-frozen and stored at -80°C until extraction of nucleic acids.

### 2.2. RNA Extraction of Tissues Samples

For RNA extraction, ileal and caecal samples from 10 high (mean RFI = 1604 g/day) and 10 low (mean RFI = −515 g/day) RFI pigs were selected. The selection of pigs was balanced for litter origin and sex. The frozen tissue was pulverized with mortar and pestle, and RNA extraction was performed with TRI reagent (Sigma-Aldrich, Taufkirchen, Germany). After this initial RNA extraction according to the manufacturer’s instructions, a DNase digestion and subsequent RNA purification was performed using DNaseI (Roche, Mannheim, Germany) and a column-based RNA extraction kit (NucleoSpin RNAII, Macherey-Nagel, Düren, Germany). For quality and quantity assessment, the extracted RNA was separated on agarose gel to check integrity, and was measured using the NanoDrop ND-2000 (Thermo Fisher Scientific, Dreieich, Germany).

### 2.3. RNA Expression Microarray Preparation and Processing

RNA samples were processed using the Affymetrix WT Plus Expression Kit (Affymetrix, Santa Clara, CA, USA) following the manufacture’s recommendations. Subsequently, samples were hybridized to the snowball microarray (Affymetrix, Affymetrix, Santa Clara, CA, USA), which contains 47,845 probes specific for the porcine transcriptome [16]. Microarrays were scanned with the Affymetrix GeneChip Scanner 3000 (Affymetrix, Affymetrix, Santa Clara, CA, USA). The probe-to-probeset assignment and corresponding annotation information for the snowball array were generated using the workflow according to Hadlich et al. [17]. Therefore, genomic information of the *Sus Scrofa* 11.1 genome map was retrieved from Ensembl (release 93) and NCBI (both accessed in August 2018) to create a custom layout file (cdf file).

An initial quality assessment (R package arrayQualityMetrics) of the intensity data was applied. The raw intensity data was normalized using the robust multichip average approach implemented in the affy R package. In order to increase the predictive capacity, inappropriate probe-sets were excluded based on mean and standard deviation. Specifically, this refers to probe-sets with a mean less than the 5% quantile among all samples and those having a standard deviation of more than the 95% quantile within each experimental group. Raw data is accessible at the National Center for Biotechnology Information Gene Expression Omnibus database (available at: www.ncbi.nlm.nih.gov/geo) (accession numbers: GSE142438).

### 2.4. 16S rRNA Amplicon Sequencing and Metagenomics Prediction

Complementary data for ileal and ceacal microbiota composition based on the V3-V4 region of the 16S rRNA sequence were taken from a previous study [15]. Raw sequence data are available in the European Nucleotide Archive (PRJEB22209). Of the 20 samples per intestinal segment, four ileum (two high RFI and two low RFI) and three caecum samples (one high RFI and two low RFI) were not included in the analysis due to the unavailability of sample material or failure of DNA extraction. Data processing followed the Quantitative Insights Into Microbial Ecology (QIIME) pipeline (version 1.9.1, available at: http://qiime.org/). The split libraries script was used with default parameters to check quality of sequences. The sequences were cleaned for chimeras with USEARCH (version 7, available at: https://www.drive5.com/usearch). Operational taxonomic units (OTU) were deduced based on 97% sequence homology using the de novo OTU picking approach. Data was summarized at phylum, family and genus level using the SILVA 16S specific database (version 111, available at: https://www.arb-silva.de). In addition, the PICRUSt software was used to predict metagenomes based on the normalized OTU tables and to subsequently categorize their putative functions [18]. Therefore, the Kyoto Encyclopedia of Genes and Genomes (KEGG) and Clusters of Orthologous Groups of proteins (COGs) databases were employed. Functions, which showed a low relative abundance (<0.01 %) among all samples of an intestinal section were excluded from further analysis.

### 2.5. Statistical Analysis

Microarray data was analyzed using the limma R package. The statistical model accounted for experimental factors, including the effect of sow (also representing the pen as experimental unit) and slaughter batch. Contrasts were retrieved based on RFI classification. Exclusively probesets with a *p*-value < 0.05 were considered as significantly differentially abundant and used for downstream data integration. Initial evaluation of genes was performed using ingenuity pathway analysis (IPA) to derive involved canonical pathways. Pathways with a *p*-value of less than 0.05 were reported excluding pathways of the IPA ‘cancer’ category.

To identify differentially abundant taxa between high and low RFI groups, data from 16S rRNA sequencing were analyzed at genus level using the DESeq2 package in the R environment. Very low abundant taxa, which had less than two observations in more than half of the samples, were excluded. Sow was included as a fixed effect in the statistical model. Differences in genera abundance with an adjusted *p*-value < 0.05 and < 0.1 were considered as significant and tending to be significant, respectively.

Data integration was conducted for the two intestinal sites by combining expression values of differentially abundant genes and the composition of the commensal microbiota at genus level employing the sparse partial least squares (sPLS) regression approach embedded in R package ‘mixOmics’ (version 6.6.2, available at: http://mixomics.org) [19]. The sPLS regression analysis showed correlations between the microbial composition and differential gene expression in order to derive interrelations in the context of divergent RFI. Initially, very low abundant taxa were removed from the microbial dataset as described above and significantly differentially abundant genes were selected from the RNA expression dataset. Subsequently, the two datasets were transformed with centered log ratio (CLR) transformation. Based on the results of sPLS regression, the first two components were selected. In each component 100 genes and 10 taxa were visualized in a clustered image map. Functional categories based on predicted metagenomic profiles were compared between low and high RFI groups using the Welch’s t-test implemented in STAMP version 2.1.3 [20].

## 3. Results

**Figure 1:**
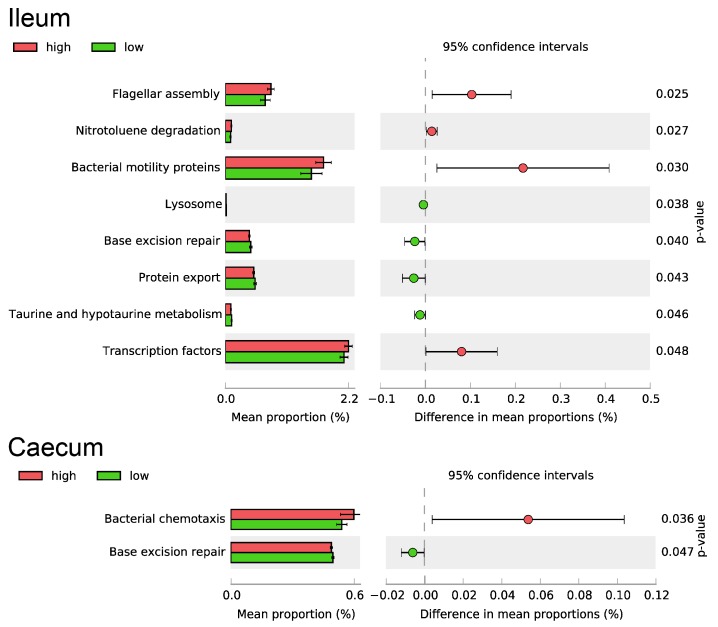
Differences in predicted functionality of ileal and caecal microbiota between pigs with high (red) and low (green) residual feed intake (RFI). Functional assignments were performed using PICRUSt by employing the Kyoto Encyclopedia of Genes and Genomes (KEGG) and Clusters of Orthologous Groups of proteins (COGs) databases.

The microbial community analysis revealed differences at the genus level between high and low RFI animals only for taxa with very low abundance (Table 1). Out of the 93 genera included in the analysis of ileum, five were found to differ significantly between groups. For caecum samples, six out of 135 genera were found to be differentially abundant between experimental groups, with all six genera having a higher relative abundance in the low RFI group compared to high RFI pigs. For ileum, the differences in abundance were most prominent for *Saccharopolyspora* and *Rothia*. Functional prediction analysis showed minor differences in flagella assembly and motility protein abundance between experimental groups (Figure 1). Regarding the metabolic profile of the ileal microbial community, differences were solely observed for nitrotoluene degradation and taurine and hypotaurine metabolism. For the caecum, OTUs assigned to the unidentified rumen bacterium RFN43 genus showed the highest significant difference in relative abundance between groups (Table 1). Additionally, *Leeia* was found to be differentially abundant in the context of divergent RFI and represented the genus with the highest relative abundance among the differentially abundant taxa identified in both intestinal sites. Based on functional prediction analysis, the bacterial movement, represented by chemotaxis, was down regulated in the caecal digesta samples from the low RFI group (Figure 1). In both intestinal sites the microbial community analysis revealed a slight increase in functions dedicated to DNA repair mechanisms in low compared to high RFI pigs.

The analysis of transcript abundance between high and low RFI pigs revealed 1171 (representing 965 annotated genes) and 1231 (representing 1007 annotated genes) differentially abundant probesets in ileum and caecum mucosa, respectively. In the ileum, two of the most prominent canonical pathways, according to IPA analysis (Supplementary Table S1), were related to metabolism and represent amino acid degradation (‘4-hydroxyproline Degradation I’) and polysaccharide biosynthesis (‘Chondroitin and Dermatan Biosynthesis’). In addition, differentially abundant genes were assigned to cellular stress and growth as well as cellular immune response pathways. For caecum mucosa, enriched canonical pathways in the context of divergent feed efficiency were mainly assigned to immune response including cellular (Th1 pathway, phagocytosis in macrophages and monocytes) and humoral (Th2, complement system) aspects (Supplementary Table S1). Specifically, the complement system pathway was predicted to be significantly activated in low RFI pigs. Other enriched pathways comprised signaling themes (i.e., ‘Integrin Signalling’) as well as the metabolism of carbohydrates and nitrogen compounds.

**Figure 2:**
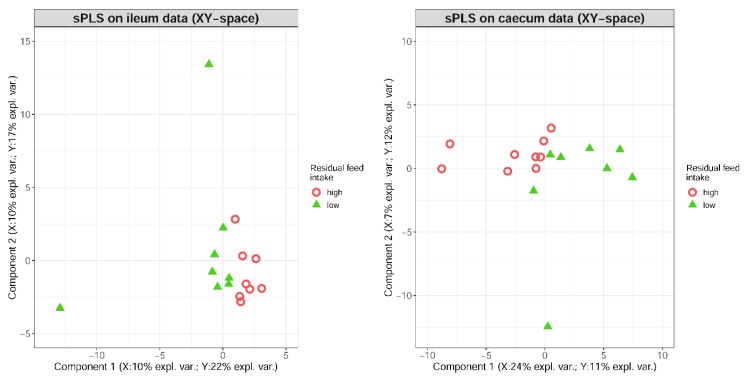
Differentiation of high (red circles) and low (green triangles) residual feed intake samples in ileum (left panel) and caecum tissues (right panel) using sparse partial least squares (sPLS). The bioinformatics approach considers the selection of 100 genes (dataset X) and 10 microbes (dataset Y) per component.

**Figure 3:**
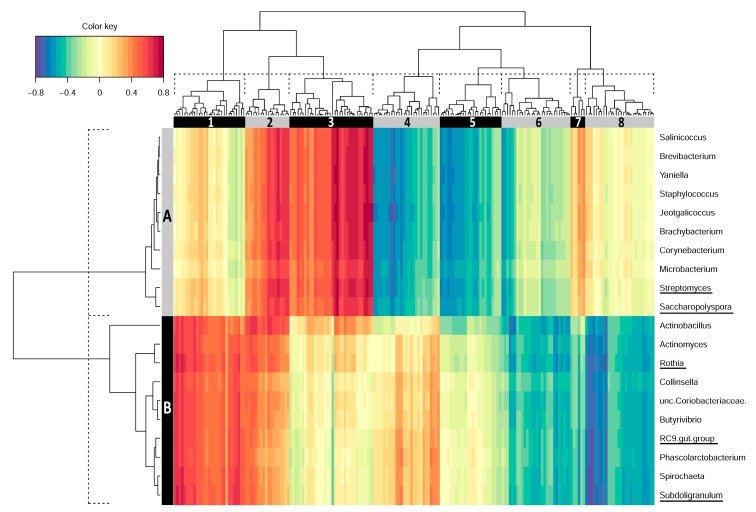
Clustered image map representing correlations between the abundance of genera in ileal digesta and differentially expressed genes of ileal mucosa in the context of divergent feed efficiency. Displayed are the top 100 genes and top 10 taxa from each of the first two components selected using a sparse partial least squares (sPLS) regression. Microbe (**A** and **B**) and gene (1–8) clusters were derived from dendrograms (dashed line) and are indicated by boxes on the top and left of the plot. Underlined taxa were differentially abundant between feed efficiency divergent pigs. The assignment of genes to the individual clusters is given in Supplementary Table S2.

Data integration was performed between the set of genes differentially expressed in RFI-divergent pigs and the abundant genera of the two intestinal sites. Based on the transcriptional and microbial variables selected by the sPLS approach, the samples of the low and high RFI groups could be differentiated for the two intestinal sites analyzed (Figure 2). The hierarchical cluster analysis based on the variables selected revealed two dominant microbial clusters for ileum samples (cluster A and B, Figure 3). With respect to the identified differentially abundant genera, cluster A contains the taxa *Saccharopolyspora* and *Streptomycetes* and cluster B is represented by *Rothia* and RC9 gut group. In total, eight gene clusters (1–8) were highlighted for ileum, whose expression values were correlated with the presence or absence of specific genera. The composition of these gene clusters and putatively affected canonical pathways are provided in Supplementary Table S2. For the first ileal microbial cluster (A), positive correlations (indicated by red color) were identified for gene clusters 2, 3, and 7. Pathway analysis of genes involved in these clusters revealed roles in DNA repair and immune cell signaling. Negative correlations of cluster A (indicated by blue color) were observed with ileal gene clusters 4, 5, and 6, whose genes are enriched in signaling pathways involved in growth processes and cellular stress. Microbial cluster B showed the strongest positive and negative correlations with gene clusters 1 and 2, and 6, 7, and 8, respectively (Figure 3). The former gene clusters also showed enrichment in DNA repair pathways and the latter in signaling transduction.

**Figure 4:**
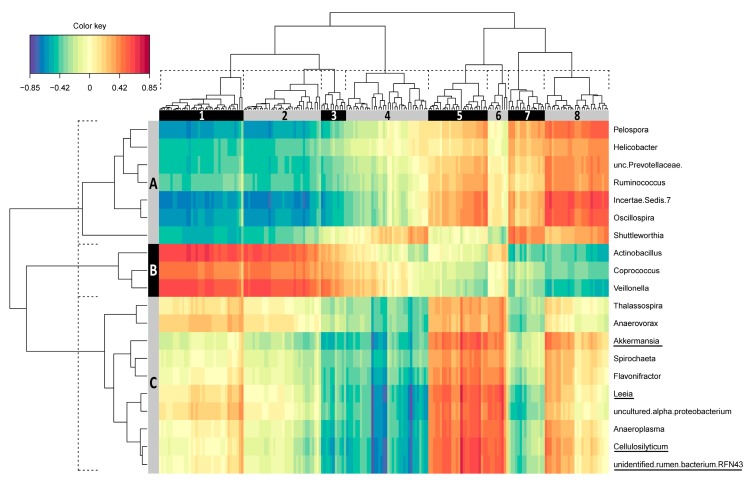
Correlations between differentially expressed mucosal genes and digesta microbe abundance of caecum in the context of divergent residual feed intake. Shown are the top 100 genes and top 10 taxa from each of the first two components selected using a sparse partial least squares (sPLS) regression. Clusters of microbes (**A**–**C**) and genes (1–8) were derived from dendrograms (dashed line) and are indicated by boxes on the top and left of the plot. Underlined taxa were differentially abundant between feed efficiency divergent pigs. The assignment of genes to the individual clusters is given in Supplementary Table S2.

For the integration of caecum data, three microbial clusters (A, B and C) and eight gene clusters (1–8) were identified (Figure 4). Genes represented by clusters 1, 2, and 3 were positively correlated with microbial cluster B and negatively correlated with microbial cluster A. Pathway analysis revealed involvement of the corresponding genes in pathways of autophagy, cell-cycle control, and immune response. Taxa, which were found to be differentially abundant in the context of divergent RFI were mainly represented by microbial cluster C. This cluster showed the strongest positive correlation with gene clusters 5 and 6, found to be enriched in immune pathways.

## 4. Discussion

The feeding efficiency of pigs, i.e., the efficient conversion of nutrients into body weight gain, has improved considerably in recent decades. This is mainly due to advances in husbandry, genetics, and feeding strategies, including the use of highly concentrated, low-fiber feed largely resembling the human Western diet. However, even when environmental conditions and genetic origin are controlled, there is still considerable variation in the feed efficiency of pigs [5,21,22,23], which were examined in this study at the host and microbiota level. One of the factors that might explain this are differences in the intestinal microbiota, whereby its composition and function can vary greatly depending on the intestinal segment and the age of the pig [3]. The current study focused on feed efficiency-related differences in host transcriptomics and the microbial composition in the ileum and caecum. In fact, the phenotype of the RFI-divergent groups was affected by alterations of biofunctions related to immunity, which were consistently associated with divergent feed efficiency at both the gene and the microbiota level, and has previously been described to contribute to improved feed efficiency [24,25]. In ileum, the results point to an altered immune competence at the border between host and microbiota, which is represented by several aspects: (i) the enrichment of the ‘Crosstalk between Dendritic Cells and Natural Killer Cells’ pathway; (ii) an altered metabolism of 4-hydroxyproline, which is part of the C1q and thus might affect the complement system; (iii) the CCR5 pathway contributing to the regulation of leukocyte chemotaxis in inflammation; and (iv) nNOS signaling, which might alter local blood flow and muscle contraction. Summarizing the results of caecum analysis, the main themes altered comprise humoral and cellular immune response engaging the complement system, Th1 and Th2 pathways and the process of phagocytosis. In particular, the general immunity represented by the complement system, appears to be upregulated in low RFI pigs. Consistently, Mani et al. observed lower serum endotoxin and haptoglobin concentrations as well as lower levels of intestinal inflammatory markers in low RFI pigs [25]. The enrichment of immune-related pathways in the ileum and caecum mucosa is coherent with transcriptional responses identified in other sites of the gastrointestinal tract when high and low RFI pigs are compared [26]. This might suggest energy saving mechanisms at the level of the intestinal immune system contributing to divergent feed efficiency [27]. Moreover, the microbial cluster A, identified in the integrative analysis of the ileum transcriptome and microbial composition, was represented by *Saccharopolyspora* and *Streptomyces*, which were also differentially abundant between high and low RFI animals. Species assigned to these genera are known to produce antibacterials [28,29]. Correlated gene clusters were enriched for immune cell signaling, which again indicates active host-microbiota interactions in terms of immune competence.

Considering the high energy concentration and low fiber content of commercial pig diets, it is expected that the main turnover of these diets takes place in the proximal parts of the gut [30,31]. Accordingly, the composition of the intestinal content, which remains after passing the proximal intestine, is likely to be of limited complexity regarding microbially digestible material, thereby affecting the microbial colonization and diversity in the ileum and caecum. This will influence the quantity of nutrients provided by ileal and caecal fiber digestion or the turnover of non-starch containing substances [31,32]. Low RFI pigs were reported to have increased ileal digestibility compared to high RFI counterparts [33]. Moreover, higher volatile fatty acid concentrations were found in the feces of low RFI pigs, compared to that of high RFI pigs [2]. The microbial cluster B of the ileum comprises genera with distinct metabolic activities, which might contribute to increased ileal digestibility in the low RFI group. Specifically, various *Rothia* species support the digestion of gluten proteins from feedstuffs [34], *Subdoligranulum* species contribute to the conversion of lactate to butyrate [35] and the RC9 gut group has been proposed as part of the core microbiota [36], associated with the turnover of starch and non-starch polysaccharide containing substances [37].

Of the taxa that were differentially abundant in the caecum of RFI-divergent pigs, *Leeia* had the highest relative abundance, representing almost 1% of the genera found. *Leeia* is a genus of the class betaproteobacteria, which has a higher abundance in the caecum compared to colon and ileum digesta [5]. Interestingly, in relation to different dietary fiber components, *Leeia* was shown to be more abundant under pectin supplementation [32]. Similarly, species of the genus *Cellulosilyticum* are known to act as cellulose-degrading bacteria [38], and the abundance of the unidentified rumen bacterium RFN43 varied with changing forage to concentrate ratios in cattle [39]. Overall, the specific shifts towards species of *Rothia*, *Subdoligranulu, Leeia, Cellulosilyticum*, and the unidentified rumen bacterium RFN43 indicate the establishment of a microbial profile that improves the digestion of dietary components which are undigested or poorly digested by endogenous enzymes in the pig. As a consequence, this increases nutrient digestion and utilization in low RFI pigs. The complementary expression analysis of ileum and caecum mucosa revealed minor shifts at the transcriptional level between pigs with divergent feed efficiency. Subsequent enrichment analysis pointed out canonical pathways of cell signaling and metabolism of certain substance classes as affected. However, major changes at the level of micro- and macronutrient transport and metabolism, which represent an essential function of the gastrointestinal tract towards improved feed efficiency, were almost absent. With regards to the functional anatomy of the gut, this is related to the fact that major genes driving absorption processes for anions, sodium, carbohydrates, amino acids, minerals, and lipids are predominately expressed in the proximal part of the intestinal tract [40]. Accordingly, previous analysis of different parts of the gastrointestinal tract and their morphology revealed no significant differences in the ileum and caecum between high and low RFI pigs, whereas some distinct differences were observed in the duodenum and jejunum, such as Goblet cell number per villus height and nutrient transporter gene expression [33,41].

## 5. Conclusions

The microbial composition and host expression in the ileum and caecum provide small impacts on feed efficiency under high concentration low fiber dietary conditions. Due to an increased abundance of non-starch polysaccharide fermenting taxa, pigs with a low RFI might be more efficient in using feed components that resisted digestion in anterior intestinal segments. Furthermore, in line with previous results, the involvement of general immunity pathways in low RFI pigs probably benefits feed efficiency through energy-saving mechanisms. Future work should determine whether dietary manipulation with prebiotics or direct fed microbials/probiotics can promote the beneficial functions observed here and be used as nutritional strategies to improve feed efficiency in pigs.

## Figures and Tables

**Table 1:** Differentially abundant genera in the ileum and caecum digesta of pigs divergent for residual feed intake (RFI).

		Relative Abundance		
Genus	Phylum	High RFI	low RFI	*p*-value
Ileum				
*Saccharopolyspora*	Actinobacteria	0.0015%	0.0287%	**0.0066**
*Rothia*	Actinobacteria	0.0119%	0.0692%	**0.0066**
*RC9 gut group*	Bacteroidetes	0.0339%	0.2150%	**0.0316**
*Streptomyces*	Actinobacteria	0.0111%	0.0482%	**0.0316**
*Uncultured Bacillaceae*	Firmicutes	0.0064%	0.0171%	**0.0316**
*Subdoligranulum*	Firmicutes	0.0181%	0.1393%	0.0780
*Uncultured S24-7*	Bacteroidetes	0.0458%	0.1059%	0.0780
*Asteroleplasma*	Firmicutes	0.0070%	0.0578%	0.0780
*Mitsuokella*	Firmicutes	0.0114%	0.0427%	0.0855
Caecum				
*Unidentified rumen bacterium RFN43*	Bacteroidetes	0.0630%	0.2043%	**0.0064**
*Cellulosilyticum*	Firmicutes	0.0257%	0.1390%	**0.0177**
*Leeia*	Proteobacteria	0.2967%	0.9385%	**0.0177**
*Akkermansia*	Verrucomicrobia	0.0096%	0.0488%	**0.0177**
*Uncultured GR.WP33.58*	Proteobacteria	0.0633%	0.0925%	**0.0177**
*Uncultured rumen bacterium*	Cyanobacteria	0.0029%	0.0176%	**0.0459**

*p*-values < 0.05 are in bold.

## Supplementary Materials

The following are available online at https://www.mdpi.com/2076-2607/8/4/563/s1, Table S1: Enriched canonical pathways among the differentially abundant genes in ileum and caecum mucosa of pigs differing in feed efficiency, Table S2: Composition of ileal and caecal gene clusters deduced from correlation analysis between differentially expressed mucosal genes and digesta microbe abundance. The enrichment of genes in canonical pathways was analyzed using Ingenuity Pathway Analysis.
